# YARG: A repository for arsenic-related genes in yeast

**DOI:** 10.1371/journal.pone.0201204

**Published:** 2018-07-26

**Authors:** Jagat Rathod, Hao-Ping Tu, Yung-I Chang, Yu-Han Chu, Yan-Yuan Tseng, Jiin-Shuh Jean, Wei-Sheng Wu

**Affiliations:** 1 Department of Earth Sciences, National Cheng Kung University, Tainan, Taiwan; 2 Department of Electrical Engineering, National Cheng Kung University, Tainan, Taiwan; 3 Center for Molecular Medicine and Genetics, Wayne State University School of Medicine, Detroit, MI, United States of America; CNR, ITALY

## Abstract

Arsenic is a toxic metalloid. Moderate levels of arsenic exposure from drinking water can cause various human health problems such as skin lesions, circulatory disorders and cancers. Thus, arsenic toxicity is a key focus area for environmental and toxicological investigations. Many arsenic-related genes in yeast have been identified by experimental strategies such as phenotypic screening and transcriptional profiling. These identified arsenic-related genes are valuable information for studying arsenic toxicity. However, the literature about these identified arsenic-related genes is widely dispersed and cannot be easily acquired by researchers. This prompts us to develop YARG (Yeast Arsenic-Related Genes) database, which comprehensively collects 3396 arsenic-related genes in the literature. For each arsenic-related gene, the number and types of experimental evidence (phenotypic screening and/or transcriptional profiling) are provided. Users can use both search and browse modes to query arsenic-related genes in YARG. We used two case studies to show that YARG can return biologically meaningful arsenic-related information for the query gene(s). We believe that YARG is a useful resource for arsenic toxicity research. YARG is available at http://cosbi4.ee.ncku.edu.tw/YARG/.

## Introduction

Arsenic (As), the 20^th^ most abundant element on earth, is a toxic metalloid. In nature, arsenic is found in two chemical forms: inorganic species [arsenite (As^3+^) and arsenate (As^5+^)] and organic species [monomethylarsonic acid (MMA) and dimethylarsinic acid (DMA)]. Inorganic forms of arsenic are more toxic than organic forms. It is well reported that arsenic affects almost all cellular processes and organ functions that manifest due to cellular stress, mitochondrial and oxidative damage, genetic mutations and epigenetic dysregulation [1]. Low to moderate levels of arsenic exposure (10–300 μg L^-1^) from drinking water can cause health problems such as skin lesions, circulatory disorders, neurological complications, diabetes, respiratory complications, hepatic and renal dysfunction [2]. Thus, arsenic toxicity is a major concern for environmental and toxicological investigations worldwide.

The availability of complete genome sequence and mutant libraries of *Saccharomyces cerevisiae* provides multi-directional opportunities to design insightful experiments to understand arsenic metabolism, detoxification and tolerance acquisition mechanisms, which are mostly conserved from yeast to human [3–5]. Majority of yeast genes involved in arsenic response have homolog in humans that could potentially modulate toxicity in a similar manner as their yeast counterparts. Therefore, the budding yeast *S*. *cerevisiae* is a useful eukaryotic model organism for studying arsenic toxicity in human health and diseases [6].

To understand how yeast cells respond to arsenic exposure, it is crucial to identify arsenic-related genes. Two main experimental strategies are used to identify arsenic-related genes systematically. The first is phenotypic screening of the *S*. *cerevisiae* mutant libraries under arsenic exposure [7–14]. Comparing growth kinetics of wild-type (WT) and a homozygous deletion mutant of a specific gene, both with and without arsenic exposure, identifies the potential role of that gene in arsenic sensitivity or resistance if it significantly affects the growth rate. The phenotypic screening is done for all ~4870 non-essential genes in the yeast genome [14]. Although most of the phenotypic screens are performed in nutrient rich media, different studies used different strains and exposed cells to various arsenic concentrations for different durations. Therefore, diverse phenotypic screenings have identified different sets of arsenic-related genes [7–14]. Phenotypic screenings supported with additional physiological and biochemical characterizations have highlighted the genetic determinants of arsenic susceptibility and resistance along with their designated functions in yeast. On a larger scale, it is known that arsenic (specifically arsenite) binds to α-helices affecting secondary structures of proteins, which could be corroborated by phenotypic screens suggesting functional inhibition of the chaperonin complex [13]. These details are critical to understand the arsenic molecular response mechanism in yeast.

The second strategy is genome-wide transcriptional profiling [7, 9, 15]. By comparing the genome-wide gene expression patterns between WT cells with and without arsenic exposure, the genes whose expressions are significantly affected by arsenic can be identified. By knowing differentially expressed genes under arsenic exposure, multiple inferences could be derived on the functioning of genetic networks, their response directions and diverse pathways involved. Systematically, yeast has evolved various defence strategies to tolerate and detoxify arsenic by reduction of metal uptake, enhanced extrusion, sequestration within vacuoles and chelation by metal-binding proteins and polypeptides [9, 15, 16]. Considering the need for a systematic and prompt response in yeast, signal transduction pathways undergo a rapid re-programming of cellular transcriptome that eventually modulates proteome and metabolome profiles. The functionality of differentially expressed genes in the defense against the arsenic-toxicity could identify a novel responsive candidate that might be critical for arsenic resistance and cellular processing. Thus, it is essential to compile and compare all global transcriptome profiling data obtained under arsenic exposure.

Further, experimental evidence shows that arsenic can directly bind to and activate transcription factor Arr1 [17]. Other transcription factors such as Yap1 (a key regulator of oxidative stress response) and Rpn4 (a key regulator of proteotoxic stress response) also play a critical role in arsenic detoxification. It is known that transcription factors Arr1, Rpn4 and Yap1 confer resistance to arsenic via regulating the expression levels of diverse genes [7]. Therefore, arsenic-related genes can also be identified by comparing the genome-wide gene expression differences between WT and transcription factor mutants (*arr1Δ*, *rpn4Δ* or *yap1Δ*) both under arsenic exposure [7, 15]. Transcriptional profiling also helps to understand the role of Arr1, Rpn4 and Yap1 in arsenic resistance and their effects on genome-wide gene expression patterns.

Additionally, Haugen *et al*. [7] utilized both phenotypic screening and gene expression analysis to draw conclusion that arsenic might channel sulfur into glutathione for detoxification, lead to indirect oxidative stress by depleting glutathione pools, and alter protein turnover via arsenation of sulfhydryl groups on proteins. They also highlighted that phenotypically sensitive pathways are upstream of differentially expressed genes, suggesting that transcriptional and phenotypic profiling implicate distinct but functionally related pathways in the yeast system. Therefore, to comprehensively characterize the arsenic-related genes, we need to integrate both phenotypic screening and transcriptional profiling datasets from all available studies in the literature.

Thousands of arsenic-related genes in yeast have been identified by many experimental studies using either phenotypic screening or transcriptional profiling, or both [7–15]. However, the literature about these identified arsenic-related genes is widely dispersed and cannot be easily acquired by researchers. Therefore, there is a need of a database which comprehensively collects the arsenic-related genes from the literature and provide a dynamic interface to all information. To meet this need, we have constructed YARG (Yeast Arsenic-Related Genes) database, which collects 3396 arsenic-related genes and their experimental evidence. Users can search YARG by gene names and get the information about their arsenic-related correlation with collective evidence from phenotypic screening and/or transcriptional profiling. Besides, users can browse YARG to retrieve 20 different lists of arsenic-related genes from nine experimental studies [7–15]. The experimental strategy, experimental strain and experimental condition of these studies are also provided. In summary, YARG is a useful resource for the scientific community to investigate arsenic toxicity in yeast.

## Construction and contents

### The configuration of YARG database

**Fig 1:**
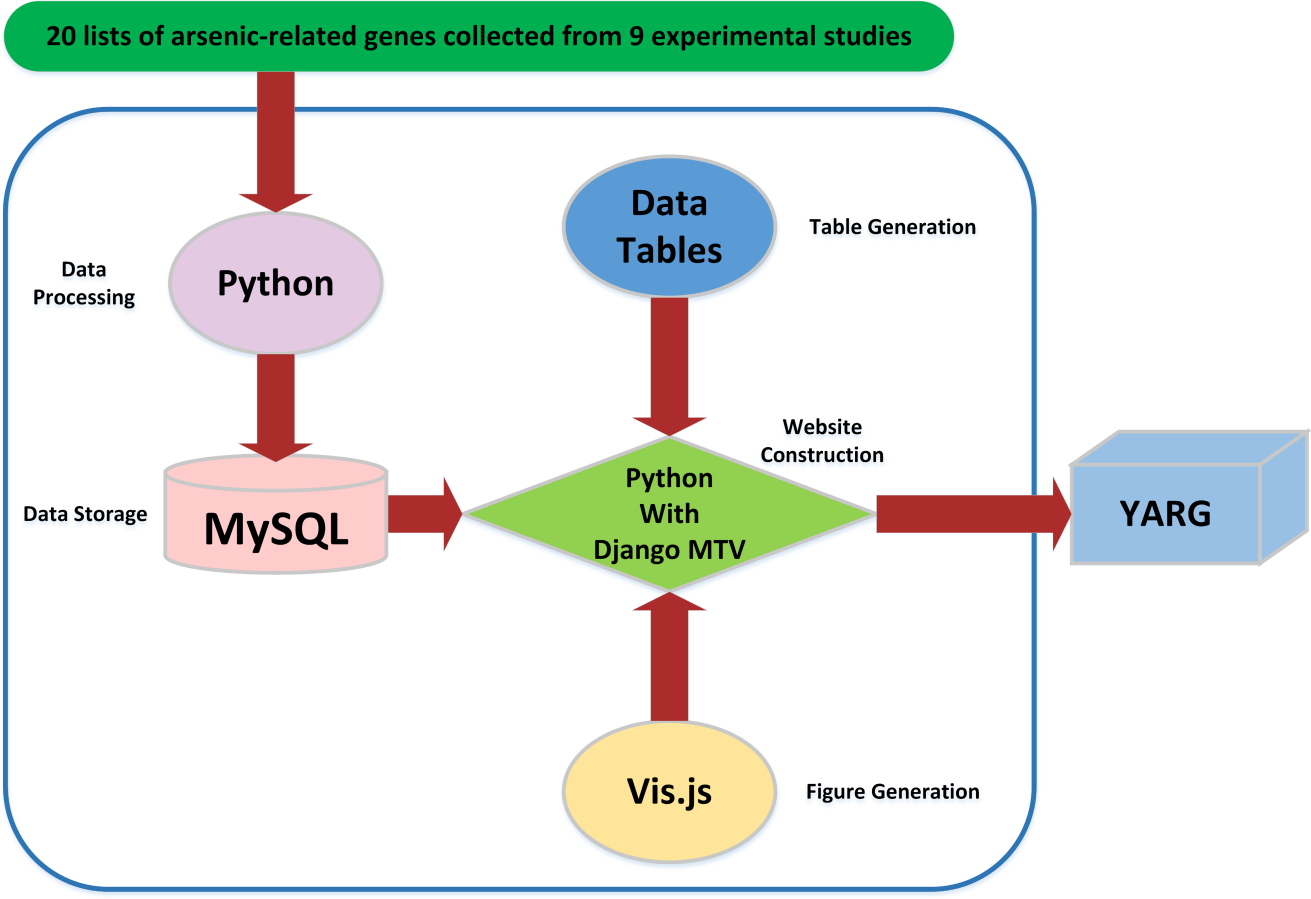
The configuration of YARG database.

Fig 1 illustrates the configuration of YARG database. Python with Django MTV framework was used to construct YARG website. Python was also used for raw data processing. The processed data was stored in MySQL. The tables were produced by Data Tables (a table plug-in for jQuery). The graphics were generated by vis.js (a browser based graphic drawing library).

### Collection of 3396 arsenic-related genes

**Fig 2:**
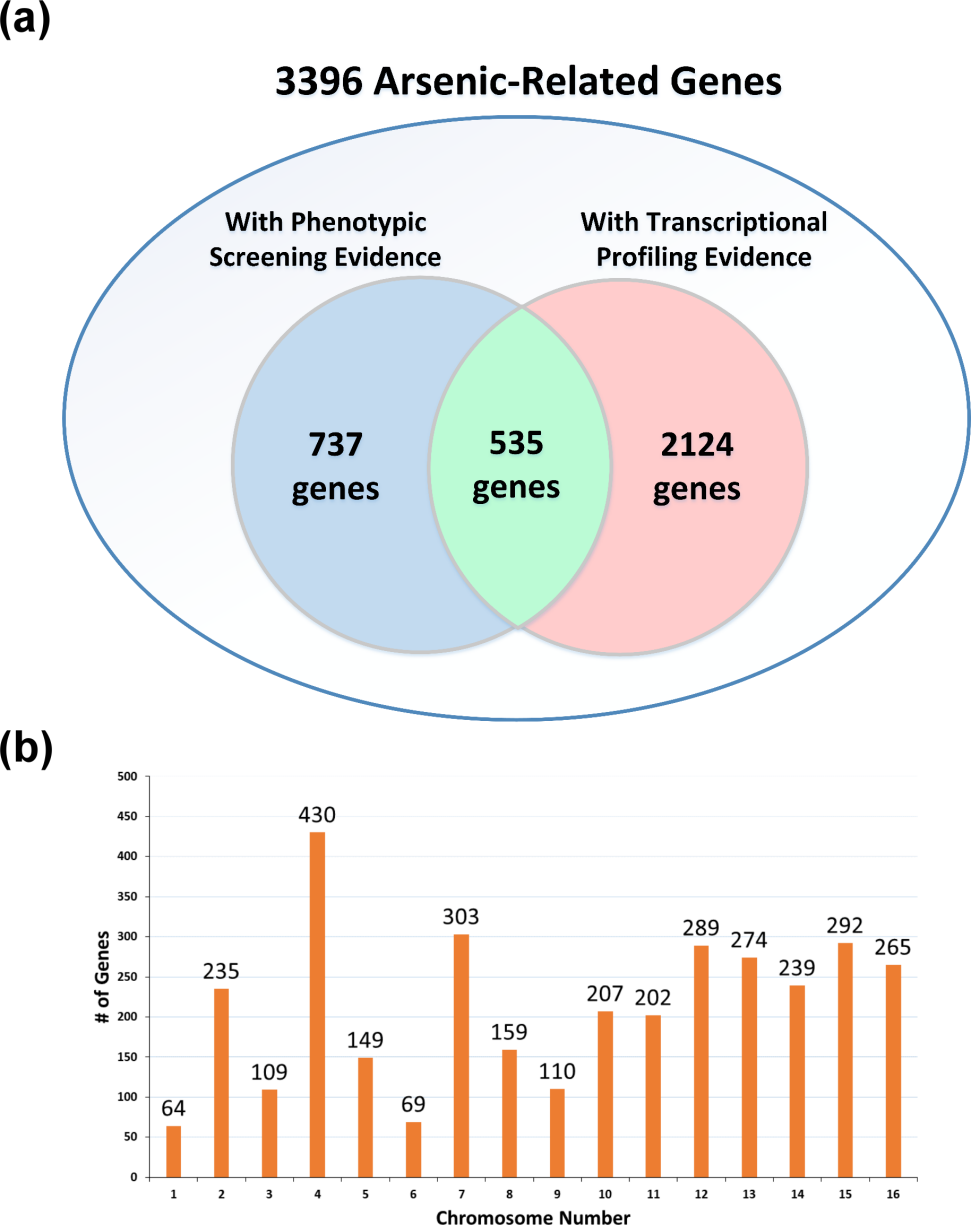
Total 3396 arsenic-related genes in YARG. (a) The 3396 arsenic-related genes are supported by phenotypic screening, transcriptional profiling, or both. (b) The distribution of the 3396 arsenic-related genes on 16 yeast chromosomes.

We collected 20 gene lists in nine existing studies [7–15] which experimentally identified arsenic-related genes by phenotypic screening (PS) or transcriptional profiling (TP). Among the 20 collected gene lists, 13 were generated by PS (Table 1) and 7 were generated by TP (Table 2). We then retrieved 3396 arsenic-related genes from these 20 collected gene lists. Among the 3396 arsenic-related genes, 535 are supported by both PS and TP, 737 are supported only by PS, and 2124 are supported only by TP (Fig 2A). The distribution of these 3396 arsenic-related genes on different chromosomes is shown in Fig 2B.

**Table 1:** Total thirteen collected lists of arsenic-related genes identified by phenotypic screening.

Source	Identified gene list	Experimental strain	Experimental growth condition (arsenic exposure)
Haugen *et al*. 2004 [7]	213 gene mutants of arsenite-sensitive phenotypes	Homozygous diploid *S*. *cerevisiae* BY4741 (*MATa his3Δ1 leu2Δ0 met15Δ0 ura3Δ0*) mutants	100 μM and 1 mM sodium arsenite for 0.5, 2 or 4 h
Vujcic *et al*. 2007 [8]	72 gene mutants of arsenite-sensitive phenotypes	*S*. *cerevisiae* BY4741 (*MATa his3Δ1 leu2Δ0 met15Δ0 ura3Δ0*) mutants	YPD plates containing sodium arsenite (2.5 or 5 mmol/L) as well as on YPD plates without any arsenic (control plates). Plates were incubated for 4 to 15 days at 30°C and phenotype of each mutant was scored as sensitive or resistant compared with control plate and internal control (BY4741 on each plate).
Jin *et al*. 2008 [9]	65 gene mutants of arsenite-sensitive phenotypes	Homozygous diploid *S*. *cerevisiae* BY4743 (*MATa/α his3Δ1/his3Δ1 leu2Δ0 /leu2Δ0 lys2Δ0/LYS2 MET15/met15Δ0 ura3Δ0 /ura3Δ0*) mutants	1.25 mM sodium arsenite for 2 h
Jo *et al*. 2009 [10]	647 gene mutants of arsenite-sensitive phenotypes	Homozygous diploid *S*. *cerevisiae* BY4743 (*MATa/α his3Δ1/his3Δ1 leu2Δ0 /leu2Δ0 lys2Δ0/LYS2 MET15/met15Δ0 ura3Δ0 /ura3Δ0*) mutants	75, 150 and 300 μM sodium arsenite for 5 and 15 generations and subsequently analysed by TAG4 arrays.
Thorsen *et al*. 2009 [11]	305 gene mutants of arsenite-sensitive phenotypes	Haploid *S*. *cerevisiae* BY4741 and homozygous diploid *S*. *cerevisiae* BY4743 mutants	0.5, 1.0 and 1.5 mM sodium arsenite for 24, 48 and 72 h
Zhou *et al*. 2009 [12]	245 gene mutants of arsenite-sensitive phenotypes	Haploid *S*. *cerevisiae* BY4741 mutants	0, 0.75 and 1 mM sodium arsenite for 60 h
Pan *et al*. 2010 [13]	191 gene mutants of arsenite-sensitive phenotypes	Heterozygous diploid *S*. *cerevisiae* BY4741 mutants	1 mM sodium arsenite for 1 h
Pan *et al*. 2010 [13]	33 gene mutants of arsenite-sensitive phenotypes	Heterozygous diploid *S*. *cerevisiae* BY4741 mutants	450 mM sodium arsenite for 10 generations
Johnson *et al*. 2016 [14]	75 gene mutants of arsenite-sensitive phenotypes	Homozygous diploid *S*. *cerevisiae* BY4743 (*MATa/α his3Δ1/his3Δ1 leu2Δ0 /leu2Δ0 lys2Δ0/LYS2 MET15/met15Δ0 ura3Δ0 /ura3Δ0*) mutants	0.2 and 0.4 mM sodium arsenite for 16 and 20 h
Zhou *et al*. 2009 [12]	5 gene mutants of arsenite-**resistant** phenotypes	Haploid *S*. *cerevisiae* BY4741 mutants	0, 0.75 and 1mM sodium arsenite for 60 h
Pan *et al*. 2010 [13]	109 gene mutants of arsenite-**resistant** phenotypes	Heterozygous diploid *S*. *cerevisiae* BY4741 mutants	1 mM sodium arsenite for 1 h
Johnson *et al*. 2016 [14]	39 gene mutants of arsenite-**resistant** phenotypes	Homozygous diploid *S*. *cerevisiae* BY4743 (*MATa/α his3Δ1/his3Δ1 leu2Δ0 /leu2Δ0 lys2Δ0/LYS2 MET15/met15Δ0 ura3Δ0 /ura3Δ0*) mutants	0.2 and 0.4 mM sodium arsenite for 16 and 20 h
Vujcic *et al*. 2007 [8]	81 gene mutants of **arsenate**-sensitive phenotypes	*S*. *cerevisiae* BY4741 and haploid *MATa* deletion mutant derived from parental strain BY4741	YPD plates containing sodium arsenate (15 or 30 mmol/L) as well as on YPD plates without any arsenic (control plates). Plates were incubated for 4 to 15 days at 30°C and phenotype of each mutant was scored as sensitive or resistant compared with control plate and internal control (BY4741 on each plate).

These 13 arsenic-related gene lists consist of 9 mutant gene lists of arsenite-sensitive phenotypes, 3 mutant gene lists of arsenite-resistant phenotypes and 1 mutant gene list of arsenate-sensitive phenotypes.

**Table 2:** Total seven collected lists of arsenic-related genes identified by transcriptional profiling.

Source	# of identified differentially expressed genes^a^	Differentially expressed when comparing	Experimental strain	Experimental growth condition (arsenic exposure)
Thorsen *et al*. 2007 [15]	756	WT 0.2 mM As(III) *vs*. WT	*S*. *cerevisiae* W303-1A	0.2 mM sodium arsenite for 1 h
Thorsen *et al*. 2007 [15]	1066	WT 1.0 mM As(III) *vs*. WT	*S*. *cerevisiae* W303-1A	1.0 mM sodium arsenite for 1 h
Jin *et al*. 2008 [9]	1194	WT 0.4 mM As(III) *vs*. WT	*S*. *cerevisiae* BY4742 (*MATα his3Δ1 leu2Δ0 lys2Δ0 ura3Δ0*)	0.4 mM sodium arsenite for 2 h
Thorsen *et al*. 2007 [15]	76	*yap1Δ* 1.0 mM As(III) *vs*. WT 1.0 mM As(III)	*S*. *cerevisiae* RW124-W303-1A *yaplΔ*::*loxP*	1.0 mM sodium arsenite for 1 h
Haugen *et al*. 2004 [7]	179	*yap1Δ* 100 μM As(III) *vs*. WT 100 μM As(III)	*yap1Δ* of *S*. *cerevisiae* BY4741 (*MATa*, *his3Δ*, *leu2Δ0*, *met15Δ0*, *uraΔ0*)	100 μM sodium arsenite for 2 h
Haugen *et al*. 2004 [7]	415	*rpn4Δ* 100 μM As(III) *vs*. WT 100 μM As(III)	*rpn4Δ S*. *cerevisiae* BY4741 (*MATa*, *his3Δ*, *leu2Δ0*, *met15Δ0*, *uraΔ0*)	100 μM sodium arsenite for 2 h
Haugen *et al*. 2004 [7]	875	*arr1Δ* 100 μM As(III) *vs*. WT 100 μM As(III)	*arr1Δ* of *S*. *cerevisiae* BY4741 (*MATa*, *his3Δ*, *leu2Δ0*, *met15Δ0*, *uraΔ0*)	100 μM sodium arsenite for 2 h

These 7 arsenic-related gene lists consist of (i) 3 lists of genes which are differentially expressed between WT and WT under arsenic exposure and (ii) 4 lists of genes which are differentially expressed between WT and the transcription factor mutants (*arr1Δ*, *rpn4Δ* or *yap1Δ*) both under arsenic exposure.

^a^A gene was considered differentially expressed if the fold-change value was greater than or equal to twofold and if the *p-value* was less than 0.001 (in [7]) or 0.01 (in [9] and [15]).

### Testing the enrichment of arsenic-related genes in input genes

For users’ input genes, YARG tests whether they are enriched with arsenic-related genes. The *p-value* is calculated using hypergeometric test [18] as follows

$$p\_value=\sum_{x\geq K}^{\min\left(A,I\right)}\frac{\left(\begin{array}{c} A\\ x \end{array})(\begin{array}{c} G-A\\ I-x \end{array}\right)}{\left(\begin{array}{c} G\\ I \end{array}\right)}$$

where *G* = 6572 is the number of genes in yeast genome, *A* = 3396 is the number of arsenic-related genes in YARG, *I* is the number of users’ input genes, and *K* is the number of input genes which are also arsenic-related genes.

## Utility and discussion

### Database interface

**Fig 3:**
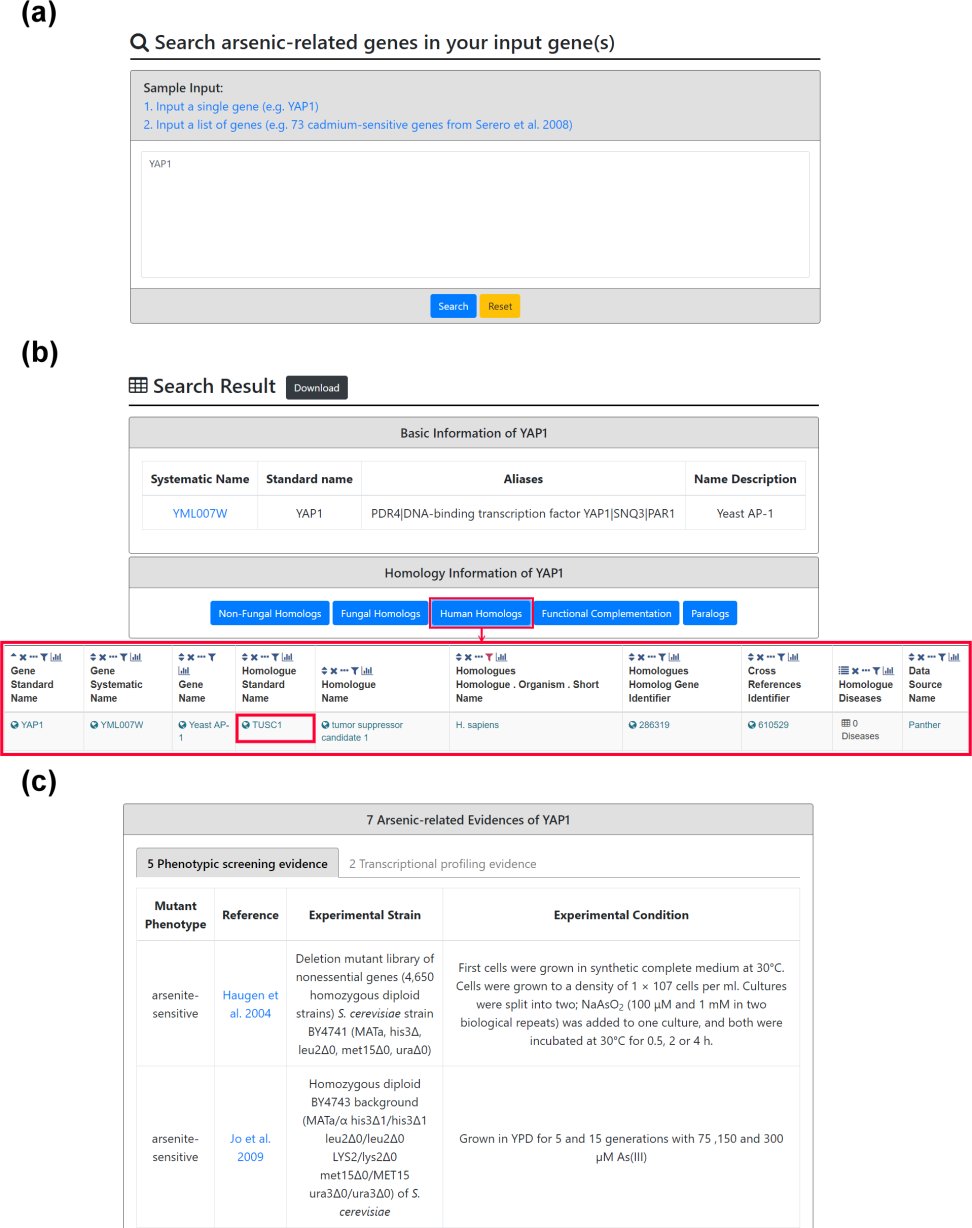
The first search mode. (a) Input a single gene name *YAP1*. (b) The basic information of *YAP1* and homology links to YeastMine. (c) Details of the arsenic-related evidence (phenotypic screening or transcriptional profiling) of *YAP1*.

**Fig 4:**
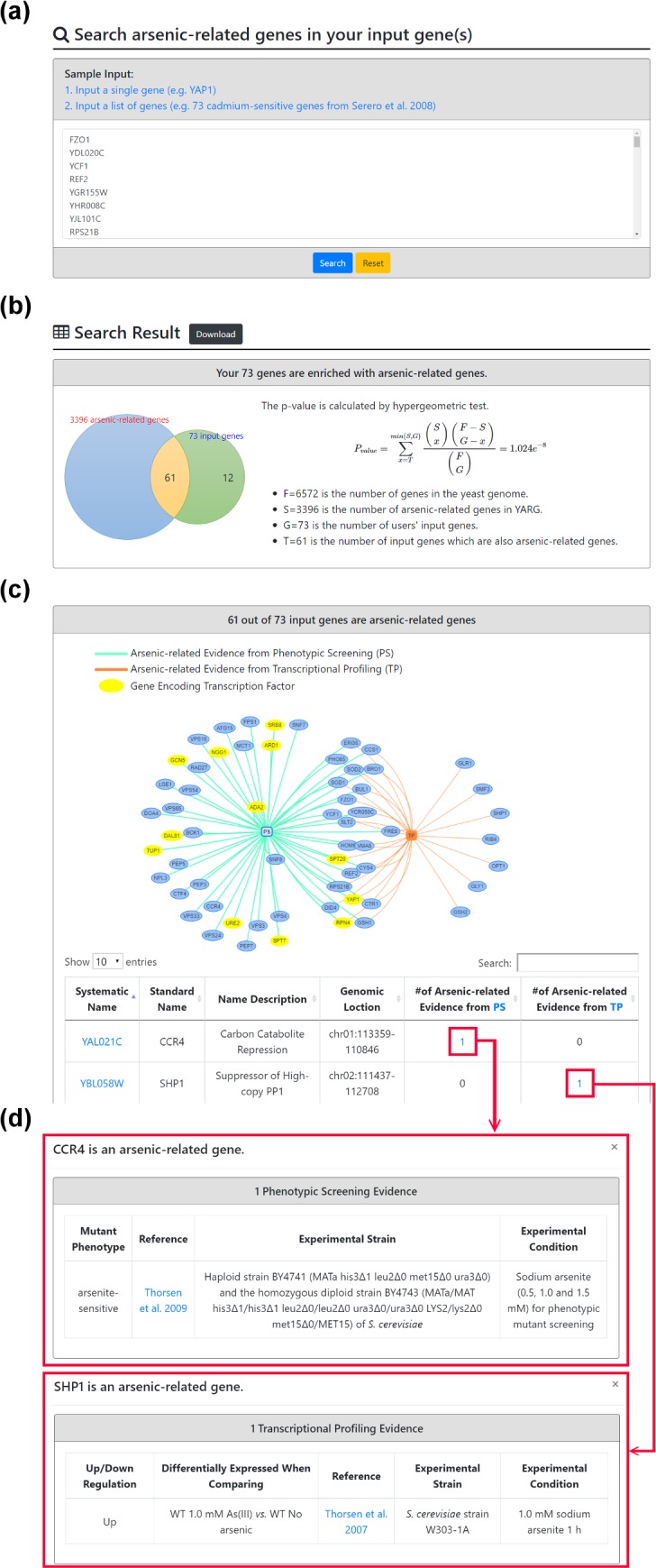
The second search mode. (a) Input a list of 73 cadmium-sensitive genes. (b) YARG tests whether the input genes are enriched with arsenic-related genes. (c) YARG provides a figure and a table to show which input genes are arsenic-related genes and the total number of supporting evidence. (d) Details of arsenic-related evidence (phenotypic screening or transcriptional profiling).

YARG provides two search modes. In the first search mode, users can input a gene name (Fig 3A). After submission, YARG returns a page showing the basic information of the input gene and links to YeastMine [19] to see the homology information such as human homologs, fungal homologs, non-fungal homologs, functional complementation and the paralogs (Fig 3B). If the input gene is an arsenic-related gene, details (experimental strain, experimental condition and reference) of the experimental evidence (phenotypic screening and/or transcriptional profiling) are provided (Fig 3C). In the second search mode, users can input a list of genes (Fig 4A). After submission, YARG uses the hypergeometric test [18] to analyze whether the input genes are enriched with arsenic-related genes (Fig 4B). YARG also provides a figure and a table to show which input genes are arsenic-related genes and total number of supporting evidences (Fig 4C). Details (experimental strain, experimental condition and reference) of the supporting evidence are also shown in Fig 4D.

**Fig 5:**
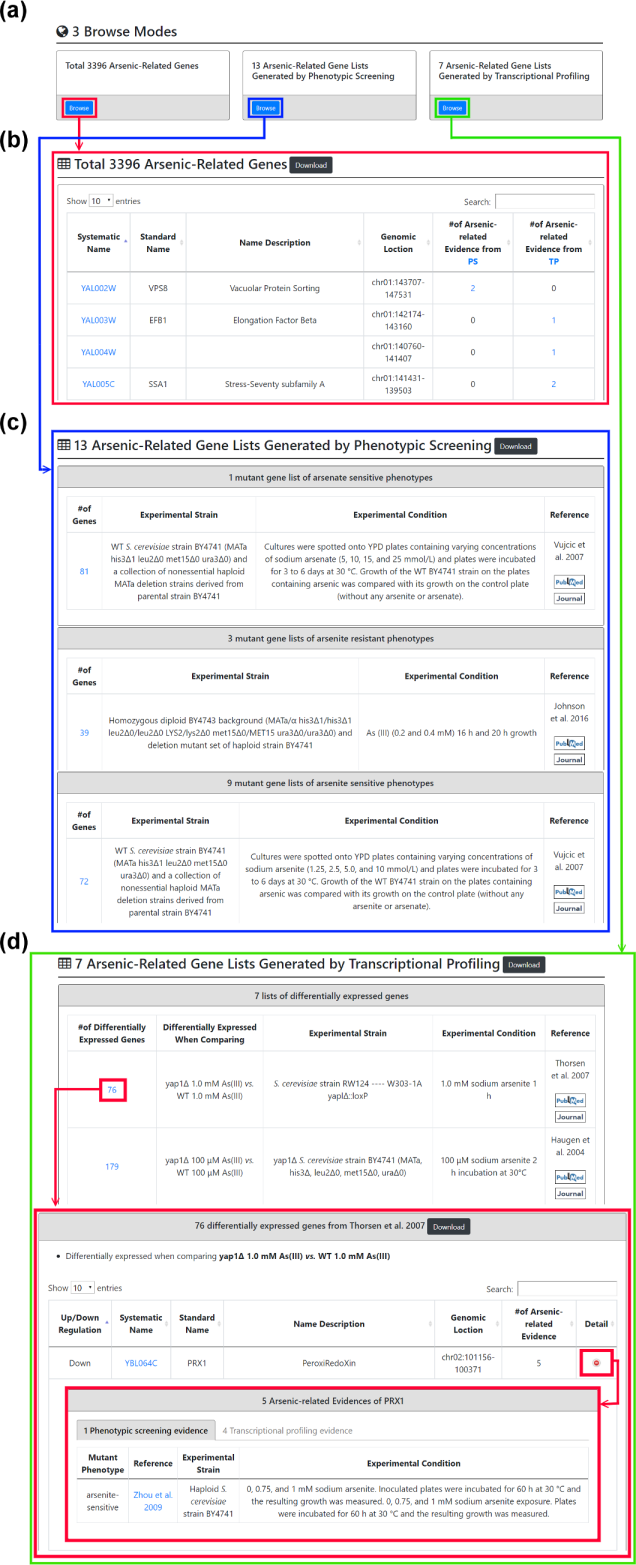
Three browse modes. (a) Three browse modes. (b) The first browse mode: users can browse 3396 arsenic-related genes in YARG. A table is given to show a systematic name, a standard name, name description, genomic location, the number of arsenic-related evidence from PS and TP. (c) The second browse mode: users can browse 13 arsenic-related gene lists generated by phenotypic screening (PS). (d) The third browse mode: users can browse 7 arsenic-related gene lists generated by transcriptional profiling (TP).

YARG provides three browse modes (Fig 5A). In the first browse mode, users can browse 3396 arsenic-related genes. For each gene, YARG provides a systematic name, a standard name, name description, genomic location, total number of arsenic-related evidence from PS and TP (Fig 5B). In the second browse mode, users can browse 13 arsenic-related gene lists generated by phenotypic screening. These 13 arsenic-related gene lists consist of 1 mutant gene list of arsenate-sensitive phenotypes, 3 mutant gene lists of arsenite-resistant phenotypes and 9 mutant gene lists of arsenite-sensitive phenotypes (Fig 5C). In the third browse mode, users can browse 7 arsenic-related gene lists generated by transcriptional profiling. These 7 arsenic-related gene lists consist of (i) 3 lists of genes which are differentially expressed between WT and WT under arsenic exposure and (ii) 4 lists of genes which are differentially expressed between WT and transcription factor mutants (*arr1Δ*, *rpn4Δ* or *yap1Δ*) both under arsenic exposure (Fig 5D).

### Two case studies

Here we give two case studies to show that the search modes of YARG can return biologically meaningful arsenic-related information for the users’ query gene(s). The first case illustrates a scenario of a single gene name submission. Yap1 is a transcription activator known to be involved in arsenic adaptation process via regulation of expression of ACR (arsenic compounds resistance) genes [17, 20, 21]. When we input a single gene name *YAP1* (Fig 3A), YARG successfully identified *YAP1* as an arsenic-related gene and provided seven arsenic-related existing experimental evidences (Fig 3C). Specifically, five phenotypic screening studies [7, 10, 11, 13, 14] utilized different experimental yeast host strains that collectively elucidate that *YAP1* is an arsenic-sensitive gene, signifying that *yap1Δ* mutant has decreased fitness and transforms host strains into arsenic-sensitive phenotype. For example, Huagen *et al*. [7] identified deletion mutants with increased sensitivity to growth inhibition utilizing an available deletion mutant library of nonessential genes (4,650 homozygous diploid strains) pinpointing that *yap1Δ* is present in the first 50 arsenic-sensitive deletion strains. Moreover, Thorsen *et al*.’s transcriptional profiling experiment [15] showed that *YAP1* is differentially expressed between wild-type strains with and without arsenic exposure. Haugen *et al*.’s transcriptional profiling experiment [7] identified 50 differentially expressed genes between wild-type and *yap1Δ* strain both under arsenic exposure. Strikingly, 20 of these 50 genes are known to play a critical role in protection against arsenic exposure, suggesting that the transcription factor Yap1 might strongly mediate arsenic-induced stress adaptation [7].

In addition to showing arsenic-related evidences of *YAP1*, YARG also provides links to YeastMine [19] for users to find out homology information of *YAP1*. For example, the human homolog(s) link to YeastMine (Fig 3B) reveals that arsenic-related gene *YAP1* has a human homolog *TUSC1* which is known to play a role in various kinds of tumorigenesis [22–25], providing a possible explanation as to why arsenic is a potential carcinogen for various kinds of cancers in human. Thus, YARG enables investigators to expose the various novel possible nexus between arsenic toxicity and human disease manifestations utilizing existing yeast toxicity studies. In summary, this case study successfully demonstrates that YARG can provide arsenic-related information and homology information for the user’s queried gene.

The second case study illustrates a scenario of a gene list submission. It is known that cadmium induces unfolded protein response, endoplasmic reticulum (ER) and oxidative stress, and hampers energy metabolism in yeast [26]. Several experimental studies have shown that *S*. *cerevisiae* uses similar detoxification mechanisms against cadmium and arsenic as other higher eukaryotic systems [9, 15, 16]. It is also well documented that the genes required for cadmium resistance have significant overlap with the genes required for arsenic resistance [27, 28]. When we input a list of 73 cadmium-sensitive genes identified by phenotypic screening from Serero *et al*. [29], YARG successfully identified that these 73 cadmium-sensitive genes are enriched (*p-value* = 1.024E-8 calculated by hypergeometric test [18]) with arsenic-related genes (Fig 4B), which are consistent with the existing knowledge [27, 28]. Specifically, 61 cadmium-sensitive genes are also arsenic-related genes with experimental evidence of phenotypic screening or/and transcriptional profiling (Fig 4C), suggesting that *S*. *cerevisiae* may use similar detoxification mechanisms against cadmium and arsenic [9, 16]. This case study clearly demonstrates that YARG can support users to compare the gene lists related to different metals and toxins, which may help in identifying novel candidate genes for toxicological research.

### Comparison with SGD and YeastMine

YARG collected 3396 arsenic-related genes supported by phenotypic screening or/and transcriptional profiling evidence from the literature. The advantages of YARG over SGD [30] and YeastMine [19] are as follows. First, SGD only allows users to check one gene at a time whether it is an arsenic-related gene according to the phenotype annotations. Second, YeastMine only allows users to retrieve a list of genes that are annotated to “metal resistance”; therefore, users still need to extract arsenic-related genes from this gene list. Third, both SGD and YeastMine define arsenic-related genes using only the phenotype annotations; neither of them provides the arsenic-related genes supported by transcriptional profiling. In summary, YARG is a useful resource of arsenic research since it provides arsenic-related genes supported by transcriptional profiling or/and phenotypic screening evidence from the literature.

## Conclusions

In this study, we present YARG, a database which is a collection of 3396 arsenic-related genes from the literature. For each arsenic-related gene, the number and types of experimental evidence (phenotypic screening and/or transcriptional profiling) are provided. Users can use both search and browse modes to query arsenic-related genes in YARG. Two case studies (a single gene *YAP1* and a list of 73 cadmium-sensitive genes) have been provided to show that YARG can retrieve biologically meaningful arsenic-related information along with experimental evidence for the users’ query gene(s). In future, we will keep updating YARG as and when new arsenic-related gene lists are available from newly published papers. YARG will be maintained regularly by our laboratory personnel. Therefore, the long-term stability of YARG is guaranteed. We also provide two backup sites (http://cosbi5.ee.ncku.edu.tw/YARG/ and http://cosbi2.ee.ncku.edu.tw/YARG/) just in case the main website is temporarily not available. We believe that YARG is a useful resource for arsenic toxicity research in yeast, supporting research community worldwide.
